# Insular Cortical Thickness Distinguishes Subjective Cognitive Decline from Normal Cognitive Aging

**DOI:** 10.1002/alz70861_108274

**Published:** 2025-12-23

**Authors:** Helen Miriam Musulan, Nhu Huynh, Susan Vandermorris, Nicolaas Paul L.G. Verhoeff, Nathan Herrmann, Linda Mah

**Affiliations:** ^1^ University of Toronto, Toronto, ON Canada; ^2^ University of Toronto, toronto, ON Canada; ^3^ Baycrest Health Science, Toronto, ON Canada; ^4^ Department of Psychiatry, Temerty Faculty of Medicine, University of Toronto, Toronto, ON Canada; ^5^ Baycrest Hospital, Toronto, ON Canada; ^6^ Rotman Research Institute, Baycrest Health Science Centre, Toronto, ON Canada; ^7^ Temerty Faculty of Medicine, University of Toronto, Toronto, ON Canada

## Abstract

**Background:**

Subjective cognitive decline (SCD), marked by memory concerns despite normal objective cognition, is considered an at‐risk group for Alzheimer’s disease (AD), but SCD due to AD is challenging to identify due to lack of objective biomarkers. This study examined group differences in cognitive performance and brain structures typically affected in AD between SCD and cognitively unimpaired (CU) older adults, and explored associations between cognition and brain structure within groups.

**Method:**

Participants included 34 SCD [17F, mean age=70.9 (5.62) years] and 26 CU [16F, mean age 71.4 (7.25) years], classified based on presence or absence of memory concerns, clinical assessment, and normal neuropsychological (NP) performance. Cognitive performance was assessed using a modified Preclinical Alzheimer’s Cognitive Composite (PACC). Structural MRI was acquired at 3T using MPRAGE. Regions‐of‐interest (ROIs) included medial temporal lobe regions, anterior and posterior cingulate, insula, and precuneus. Group differences in ROIs and PACC were tested using ANCOVA, covarying for age, sex and education. Pearson’s r was used to assess relationships between PACC scores and ROIs which differed amongst groups. Benjamini‐Hochberg was used to correct for multiple comparisons in all analyses.

**Result:**

Relative to CU, SCD participants scored lower on the PACC (F(1, 55) = 11.12, *p* = 0.00156), which remained significant after correction for multiple comparisons. Amongst ROIs, only the left insula showed a group difference, with reduced cortical thickness in SCD compared to CU (F(1, 55) = 4.81, *p* = 0.0302). Left insular cortical thickness was positively correlated with PACC scores (r = 0.34, *p* = 0.0469) in the SCD group. However, neither finding involving the left insula remained significant after Benjamini‐Hochberg correction. No association between PACC and the left insula was observed in the CU group.

**Conclusion:**

Despite normal neuropsychological test performance, individuals with SCD show lower PACC scores and reduction of left insular cortical thickness. The involvement of the insula in SCD is compatible with the higher prevalence of anxiety and depression symptoms in SCD reported in the literature. These findings require replication using larger sample sizes and AD‐related neuropathologic markers to support the potential utility of insular cortical atrophy as a biomarker for SCD due to AD.

